# Risk Factors of Incidental Parathyroidectomy and its Relationship with Hypocalcemia after Thyroidectomy: A Retrospective Study

**DOI:** 10.7759/cureus.5920

**Published:** 2019-10-16

**Authors:** Erdem Karadeniz, Mufide N Akcay

**Affiliations:** 1 General Surgery, Ataturk University, Erzurum, TUR

**Keywords:** hypocalcaemia, thyroidectomy, incidental, parathyroidectomy

## Abstract

Background: The aim of this study was to determine the incidence of incidental parathyroidectomy, the relationship between incidental parathyroidectomy and postoperative hypocalcemia, and risk factors for incidental parathyroidectomy in patients undergoing thyroid surgery.

Methods: The study was conducted by analyzing the records of patients who underwent thyroid surgery in a tertiary university hospital between January 2012 and December 2017 retrospectively. The risk factors of postoperative hypocalcemia were determined by comparing postoperative Ca values with age, sex, preoperative Ca value, dominant nodule diameter, type of surgery, and histopathological examination of the thyroidectomy material. According to the final pathology results, the patients were divided into two groups - the ones with and without incidental parathyroidectomy. The risk factors for incidental parathyroidectomy were determined by comparing the two groups in terms of age, sex, dominant nodule diameter, type of surgery, and histopathological results (malign/benign).

Results: When the risk factors of postoperative hypocalcemia were examined, female gender, age <28.5 years old, low level of preoperative mean Ca value, and total thyroidectomy were found to be critical risk factors (p<0.05). When the risk factors of incidental parathyroidectomy were examined, total thyroidectomy and thyroid malignancy were found to be important risk factors (p<0.05).

Conclusion: Female gender, age<28.5 years old, low level of preoperative Ca value, and total thyroidectomy were associated with postoperative hypocalcemia, but no relationship was found between incidental parathyroidectomy and postoperative hypocalcemia.

## Introduction

Thyroid surgeries are considered to be secure operations when they are conducted by practiced hands. Serious complications may be seen in less than 5% of the patients [1]; the frequent ones being hypocalcemia, recurrent laryngeal nerve injury, and hematoma [2]. The transient hypocalcemia rate after thyroid surgery is 27% (19-38) while permanent hypocalcemia is about 1% (0-3). Symptoms related to hypocalcemia occur in the first 24-48 hours after the surgery [3]. Hypoparathyroidism is caused by direct injury of the parathyroid gland, devascularisation, venous drainage obstruction, and accidental extraction of the parathyroid gland [4]. Although careful dissections are performed in thyroid surgery, accidental extraction of the parathyroid gland happens frequently. It is stated that the incidental parathyroidectomy rate of thyroid surgeries is between 5.2% and 21.6% [5-6]. 

The aim of this study was to determine the incidence of incidental parathyroidectomy, the relationship between incidental parathyroidectomy, postoperative hypocalcemia, and risk factors for incidental parathyroidectomy in patients undergoing thyroid surgery in our clinic.

## Materials and methods

The study was conducted by analysing the records of patients who underwent thyroid surgery in a tertiary university hospital between January 2012 and December 2017 retrospectively. Patients who had relapsed and had experienced central neck dissection were excluded from the study. Postoperative Ca values with age, sex, preoperative Ca value, dominant nodule diameter in ultrasonography (USG), type of surgery (total/lobectomy), and the results of histopathological examination of the thyroidectomy material (malign/benign, existence of incidental parathyroidectomy) were recorded. Evaluated serum calcium level under 8 mg/dL after the first 24 hours of surgery is indicated as postoperative hypocalcemia. Hypocalcemia lasting less than six months was accepted as transient hypocalcemia and hypocalcemia lasting longer than six months was accepted as permanent hypocalcemia. Vocal cord paralysis lasting less than six months was accepted as transient and vocal cord paralysis lasting longer than six months was accepted as permanent. Postoperative Ca values were compared with age, sex, preoperative Ca value, dominant nodule diameter in USG, type of surgery (total/lobectomy), and histopathological examination of the thyroidectomy material (malign/benign, existence of incidental parathyroidectomy) to determine the risk factors for postoperative hypocalcemia. In addition, according to the final pathology results, patients were divided into two groups - the ones with and without incidental parathyroidectomy. The risk factors for incidental parathyroidectomy were determined.

All collected data were analyzed using the Statistical Package for the Social Sciences software, version 20 (SPSS; IBM Corp, Armonk, NY). Data were presented as mean, standard deviation, median, minimum and maximum, percentage, and counts. When the sample size of continuous variables was <50, the Shapiro-Wilk W test was conducted; when the values were >50, the Kolmogorov-Simirnov test was conducted. When normal distribution was provided between the two independent groups, independent-samples t-test was applied; when normal distribution was not provided, the Mann-Whitney U test was performed. When the value of 2 x 2 comparisons between categorical variables were >5, the Pearson Chi-square test was applied; when the expected value was between 3 and 5, the Yates test was performed. However, when the expected value was <3, Fisher's exact test was conducted. In the multivariable analysis estimator, the risk factors between the groups were indicated through logistic regression analysis with the possible risk factors found in the previous analysis. P< 0.05 was considered statistically significant.

## Results

Out of 864 patients, 193 were males and 671 were females. Dominant nodule diameter in USG was 2.9 cm (range 0.5 - 10.5 cm). Total thyroidectomy was performed in 743 of the patients and lobectomy was performed in 121 of them. Transient postoperative hypocalcemia was seen on 268 patients (31.01%) and no persistent hypocalcemia was observed during follow-up. Transient vocal cord paralysis was seen in 15 patients (1.7%) and permanent vocal cord paralysis was observed in four patients (0.46%). Postoperative bleeding was seen in 18 patients (2%). Twelve of them underwent reoperation and bleeding control was made. Six patients were followed with drain. There was no postoperative mortality (Table 1).

**Table 1:** Demographical details of patients

	n = 864 (%)
Age	48,8 (13-91)
Gender	
Female	671 (77.7)
Male	193 (22.3)
Thyroid pathology	
Benign	557 (64.5)
Malign	307 (35.5)
Thyroid surgery type	
Lobectomy	121 (14)
Total	743 (86)
Postoperative hypocalcemia	
Transient	268 (31)
Permanent	0 (0)
Vocal cord paralysis	
Transient	15 (1.7)
Permanent	4 (0.4)
Postoperative bleeding	
Yes	18 (2)
No	846 (98)
Incidental parathyroidectomy	
Yes	80 (9.2)
No	784 (90.8)

According to postoperative histopathology results, 307 patients (35.5%) were in the malign group while 557 of them (64.5%) were placed in the benign group. Histopathologic types of malign group: 265 patients had papillary carcinoma, 17 patients had hurtle cell carcinoma, 15 patients had follicular carcinoma, five patients had medullary carcinoma, and five patients had anaplastic carcinoma. Incidental parathyroidectomy was observed in 80 (9.2%) patients in the histopathologic examination, whereas 784 (90.8%) patients did not. Six patients who experienced incidental parathyroidectomy had two parathyroid glands and 74 patients who experienced incidental parathyroidectomy had only one. Five patients (6.2%) underwent incidental parathyroidectomy in the intrathyroid gland.

In the comparison between the female and male groups, it was seen that hypocalcemia rate of females was higher than males and this difference was statistically significant (p=0.003). The evaluation of postoperative hypocalcemia in terms of age indicated that the age average of the group which suffered hypocalcemia was lower this difference was statistically significant (p=0.016). According to ROC analysis; It was found to be risky for hypocalcemia under the age of 28.5 years with %90 sensitivity . The evaluation of hypocalcemia in terms of preoperative Ca values demonstrated that transient hypocalcemia was lower in the group with higher preoperative Ca rate and this difference was statistically significant (p=0.000). In terms of the type of surgery, the transient hypocalcemia rate was higher in the group which experienced total thyroidectomy than the group which had experienced lobectomy and this difference was statistically significant (p=0.006). There was no relation between the dominant nodule diameter and hypocalcemia. When the patients were divided into malign and benign groups, no significant difference was found between these groups in terms of postoperative hypocalcemia. When the patients were divided into groups with and without incidental parathyroidectomy, no significant difference was found between these groups in terms of postoperative hypocalcemia (Table 2).

**Table 2:** Risk factors for incidental parathyroidectomy

Variables		Incidental parathyroidectomy		p
		Yes	No	
Age (average)		48.9	48.8	0.962
Gender	Female	65	606	0.409
	Male	15	178	
Thyroid surgery type	Lobectomy	6	115	0.037
	Total	77	666	
Dominant nodule diameter		3	2.8	0.435
Thyroid pathology	Benign	173	384	0.01
	Malign	95	212	

In terms of incidental parathyroidectomy risk factors, the incidental parathyroidectomy rate was higher in the group which experienced total thyroidectomy than the group which had experienced lobectomy (p=0.037). The incidental parathyroidectomy rate was higher in the malign group than in the benign group (p=0.01) (Table 3).

**Table 3:** Multivariable analysis related to postoperative hypocalcemia

Variables		Hypocalcemia		p
		Yes	No	
Age (average)		48	49	0.016
Gender	Female	225	446	0.003
	Male	42	151	
Preoperative Ca (average)		9.38	9.66	0.000
Thyroid surgery type	Lobectomy	25	96	0.006
	Total	243	500	
Dominant nodule diameter		2.81	2.87	0.109
Thyroid pathology	Benign	43	514	0.959
	Malign	40	267	
Incidental parathyroidectomy	Yes	32	48	0.069
	No	236	548	

## Discussion

Thyroid diseases have an important place among endocrine diseases. Thyroid surgeries are the most common endocrine operations that are performed [7]. The most common complication of thyroidectomy is hypocalcemia; the incidence of transient hypocalcemia is between 0.3%-49% and the incidence of permanent hypocalcemia is reported to be 0%-13% [8]. Many biochemical and harmonic tests are done due to hypocalcemia and it causes an increase in the duration of hospital stay. Hypocalcemia spontaneously resolves in many patients, but hypocalcemia can be persistent if irreversible damage occurs in the parathyroid gland. As a result of this complication, the overall cost of thyroidectomy also increases [9]. 

In our study, the rate of postoperative transient hypocalcemia was 31.01%. It was seen that this rate was a bit higher by comparison with meta-analysis of the literature (27% (19-38)) [10]. Our permanent hypocalcemia rate was lower than the rate in the literature (1%).

In terms of gender comparison, it was indicated that the hypocalcemia rate was higher in females than males. In a meta-analysis involving 10 studies and 3,443 patients, the female gender was reported as an independent risk factor for postoperative hypocalcemia [10]. In light of our study and literature information, the female gender has been seen as an independent risk factor. Recent studies on the size and anatomical localization of the parathyroid gland between men and women may explain this situation.

In our study, when the age was compared with postoperative hypocalcemia, the mean age was lower in the group with hypocalcemia. In our study; it was found that age under 28.5 years old was risky for hypocalcemia. Contrary to our study, in the literature, there are studies reporting that the mean age of the group with hypocalcemia is higher than the group without hypocalcemia [11-12]. In a meta-analysis involving five studies and 2,576 patients, age was reported as a non-risk factor for postoperative hypocalcemia [10].

In our study, preoperative Ca values were found to be lower in the patient group that developed postoperative hypocalcemia as compared to the group without postoperative hypocalcemia. In a meta-analysis involving six studies and 2,443 patients, it was reported that there was no difference in the mean preoperative Ca values when patients who developed postoperative hypocalcemia were compared with those who did not [10]. In our study, it is indicated that the postoperative hypocalcemia rate was higher in the group which underwent total thyroidectomy than the group which underwent lobectomy. In the literature, there are studies reporting that total thyroidectomy is a risk factor for postoperative hypocalcemia [11,13-14]. This can be explained by the high possibility of direct injury of glands or the vascular injury due to all the four glands on the dissection area during total thyroidectomy. In our study, no correlation was found between the dominant nodule diameter measured in preoperative USG and postoperative hypocalcemia.

In our study, no difference was found between the two groups in terms of the development of postoperative hypocalcemia when the patients were classified as malignant and benign according to histopathological results. Similar to our study, in the literature, there are studies reporting that malignancy is not a risk factor for postoperative hypocalcemia [11,13]. Although the possibility of an invasion of the malignant nodule over the surrounding tissues and parathyroid gland in malignant patients increases the risk of vascular and parenchymal injury of the parathyroid glands, no significant difference was found in our study.

In our study, no difference was found between the two groups in terms of postoperative hypocalcemia when the patients undergoing incidental parathyroidectomy were compared with those without incidental parathyroidectomy. Similar to our study, in the literature, there are studies reporting that incidental parathyroidectomy is not associated with postoperative hypocalcemia [13,15], but on the other hand, there are studies that report incidental parathyroidectomy as a risk factor for postoperative hypocalcemia [14]. A meta-analysis involving four studies and 1,482 patients reported incidental parathyroidectomy as a risk factor for postoperative hypocalcemia [10]. Hypoparathyroidism is a multifactorial situation caused by the direct injury of the parathyroid gland, devascularisation, venous drainage obstruction, and accidental extraction of the parathyroid gland. Therefore, taking only one or several parathyroid glands alone as a single parameter without considering the status of the remaining parathyroid glands and associating it with hypocalcemia may not be an appropriate assessment. In thyroid surgery, it is vital to preserve the vascular structures of the parathyroid glands by ligating the thyroid vessels closest to the thyroid capsule during the dissection of glands.

The major limitation of the study is the retrospective design of the chart. But it can be considered quite a sufficient number for a single-center series of 864 patients.

In our study, the incidental parathyroidectomy rate was found higher in the malignant patient group and the patients who underwent total thyroidectomy. A meta-analysis, as well as some studies in the literature, showed that total thyroidectomy and malignancy were the risk factors for incidental parathyroidectomy [16-18]. All four glands being present in the dissection area during the total thyroidectomy operation might explain the increase of incidental parathyroidectomy risk ratio. In the presence of malignant thyroid diseases, total thyroidectomy surgery and invasion of malignant thyroid tissue to the parathyroid gland or surrounding tissues may explain the increased risk for incidental parathyroidectomy.

## Conclusions

In our study, female gender, age<28.5 years old, preoperative low Ca values, and total thyroidectomy were indicated as risk factors for postoperative hypocalcemia. Although there is no relationship between incidental parathyroidectomy and postoperative hypocalcemia, this complication can be avoided by careful dissection and revealing glands, especially in patients undergoing malignant and total thyroidectomy.
